# The prevalence and correlates of adult separation anxiety disorder in an anxiety clinic

**DOI:** 10.1186/1471-244X-10-21

**Published:** 2010-03-10

**Authors:** Derrick M Silove, Claire L Marnane, Renate Wagner, Vijaya L Manicavasagar, Susan Rees

**Affiliations:** 1 Centre for Population Mental Health Research, Psychiatry Research and Teaching Unit, Level 1 Mental Health Centre, Liverpool Hospital, corner Forbes and Campbell St, Liverpool NSW 2170, Australia; 2 School of Psychiatry, University of New South Wales, Randwick NSW 2031, Australia; 3 Clinic for Anxiety and Traumatic Stress, Bankstown Hospital, Bankstown NSW 2200, Australia; 4 Black Dog Institute, Prince of Wales Hospital, Randwick NSW 2031, Australia

## Abstract

**Background:**

Adult separation anxiety disorder (ASAD) has been identified recently, but there is a paucity of data about its prevalence and associated characteristics amongst anxiety patients. This study assessed the prevalence and risk factor profile associated with ASAD in an anxiety clinic.

**Methods:**

Clinical psychologists assigned 520 consecutive patients to DSM-IV adult anxiety subcategories using the SCID. We also measured demographic factors and reports of early separation anxiety (the Separation Anxiety Symptom Inventory and a retrospective diagnosis of childhood separation anxiety disorder). Other self-report measures included the Adult Separation Anxiety Symptom Questionnaire (ASA-27), the Depression, Anxiety, Stress Scales (DASS-21), personality traits measured by the NEO PI-R and the Work and Social Adjustment Scale. These measures were included in three models examining for overall differences and then by gender: Model 1 compared the conventional SCID anxiety subtypes (excluding PTSD and OCD because of insufficient numbers); Model 2 divided the sample into those with and without ASAD; Model 3 compared those with ASAD with the individual anxiety subtypes in the residual group.

**Results:**

Patients with ASAD had elevated early separation anxiety scores but this association was unique in females only. Except for social phobia in relation to some comparisons, those with ASAD recorded more severe symptoms of depression, anxiety and stress, higher neuroticism scores, and greater levels of disability.

**Conclusions:**

Patients with ASAD attending an anxiety clinic are highly symptomatic and disabled. The findings have implications for the classification, clinical identification and treatment of adult anxiety disorders.

## Background

The adult form of separation anxiety disorder (ASAD) has only recently been described in the psychiatric literature [1,2]. The National Comorbidity Study Replication [3] was the first large-scale epidemiological study to include the diagnosis, revealing a lifetime prevalence of 6.6%. Apart from minor symptom differences associated with maturation, the adult pattern appears to parallel the established category of childhood separation anxiety disorder (CSAD) [1]. Affected adults experience intense fears that harm will befall close attachment figures, engaging in a range of strategies to maintain close contact with them. When faced with real or feared separations from family members, persons with ASAD are at risk of developing panic attacks [1]. Although onset can be in adulthood [3,4], in many cases early symptoms appear for the first time in childhood, persisting into the later years [2].

There is early evidence suggesting that ASAD is distinct from other adult anxiety disorders, although comorbidity is common [4]. Adult and childhood separation anxiety disorders tend to cluster in families [5], with one study suggesting an hereditary pattern, specific to females, that is distinct from neuroticism [6]. Persons with ASAD tend to report exposure to parental over-protectiveness in childhood, compared to uncaring parenting, the general pattern reported by persons with other forms of anxiety [7].

Two recent studies have investigated whether the presence of ASAD influences treatment outcomes for anxiety patients. Aaronson and colleagues [8] found that, compared to patients with panic disorder or panic disorder-agoraphobia alone, those with comorbid ASAD were 3.7 times more likely to experience a poor treatment response to cognitive behavioural therapy (CBT). Additionally, Kirsten et al [9] reported that the presence of ASAD predicted poor recovery from general symptoms of anxiety and depression amongst patients receiving CBT. It seems possible, therefore, that a failure to identify ASAD in clinic settings and to offer affected persons appropriate interventions that focus specifically on their core anxieties, may limit treatment outcomes amongst anxiety patients as a whole [10]. As yet, no specific therapies, whether psychological or pharmacological, have been devised for ASAD.

Given the recency of its identification, the diagnosis of ASAD is not widely recognised in primary care or in specialist clinics. As yet, there are limited data about the prevalence of ASAD and its correlates amongst patients referred to anxiety clinics. The present study aimed to apply a clinical research model to assess three issues amongst an anxiety clinic population, namely: 1. The prevalence of ASAD relative to other anxiety subtypes; 2. How the inclusion of the category of ASAD altered risk factor profiles across the anxiety subtypes; and 3. The level of symptom severity and functional impairment associated with ASAD.

## Methods

### Subjects

Subjects were 520 consecutive patients attending an outpatient anxiety clinic in Sydney, Australia, between 1999 and 2004. The clinic is the only public service of its kind in the catchment area, providing cost-free outpatient cognitive behavioural treatments for the full range of adult anxiety disorders. The diagnostic profile of patients attending the clinic is similar to that of comparable services in other English-speaking countries [11]. Patients in the study were mainly referred by primary care providers with non-specific diagnoses of "anxiety". Eligibility for intake is not influenced by either the duration of symptoms or history of prior treatment. At the initial intake assessment, psychologists at the clinic administered the anxiety and mood disorder modules (A and F) of the Structured Clinical Interview [SCID-I/P, [12]] to assign relevant DSM-IV-TR diagnoses. The depression module was included because of the known pattern of comorbidity within the affective disorders. Psychologists recorded all DSM-IV-TR anxiety and depressive diagnoses. If more than one disorder was identified, they used their clinical judgement to decide which disorder represented the primary problem, based on symptom severity, patient-perceived salience of the problem and associated disability. If a depressive disorder was judged to be the dominant problem, patients were referred to other relevant services. In addition, a comprehensive clinical interview was undertaken to detect other disorders such as psychosis (rarely presenting to the clinic), and if detected, these patients were referred to other services. All psychologists had received extensive training in the application of the SCID-I/P and they were required to achieve 100% inter-rater reliability with the senior clinical psychologist (at the time of the study, RW, who had over 20 years of clinical experience) prior to undertaking assessments at the clinic.

Initial examination of the data indicated relatively low numbers with a primary diagnosis of obsessive compulsive disorder (OCD, *n *= 23) and post-traumatic stress disorder (PTSD, *n *= 18). The low referral pattern for these disorders was most likely due to the availability of specialist clinics for these two conditions in Sydney. Hence, those referred to our clinic would not be typical of a help-seeking population with the relevant diagnoses, and the small cell sizes would not allow these categories to be validly included in the statistical analyses we intended to undertake. For these reasons, the categories of OCD and PTSD were excluded from further consideration in the present study. Hence, the primary DSM-IV anxiety diagnoses included in the present study were: panic disorder (PD), panic-agoraphobia (PD-AG), generalised anxiety disorder (GAD) and social phobia (SP). Comorbid mood disorders included major depressive disorder, major depressive episode and dysthymia. Because of the limited numbers assigned to each of these depressive categories, they were collapsed into a composite grouping, "current depression". Following the clinical interview undertaken at the first intake session, patients were familiarised with, and where there was a need, guided through the completion of a number of self-report questionnaires (see hereunder).

All patients signed consent forms in accordance with the ethics requirements of the Sydney South West Area Health Service.

### Measures

Modules A and F of the Structured Clinical Interview for DSM-IV-TR - SCID-I/P [12] were used. The SCID-I/P is a clinician-administered semi-structured interview for diagnosing Axis 1 disorders. Reliability coefficients from other studies have yielded kappa coefficients ranging from 0.77 to 0.95 for the relevant anxiety disorders [13].

The Adult Separation Anxiety Symptom Questionnaire - ASA-27 [14] is a 27-item self-report measure with items rated on a scale from 0 (this never happens) to 3 (this happens all the time). The psychometric characteristics of the measure have been described previously [14]. The measure has been compared with a semi-structured clinical interview (the Adult Separation Anxiety Semi-structured Interview), modelled on the SCID. A high area under the curve (AUC) value of 0.9 [14] indicated an excellent level of concordance between the two instruments.

ASAD diagnoses were based on an algorithm derived from DSM-IV-TR symptom criteria for separation anxiety disorder [15], excluding the provision that symptoms had to commence in childhood. Additional file 1 shows the items in the measure that correspond to the relevant DSM-IV-TR criteria. As an example, question 2 in the ASA-27 inquires about anxieties about leaving home, reflecting the DSM-IV-TR criterion of recurrent excessive distress when separation from home or major attachment figures occurs or is anticipated. We then applied the DSM-IV-TR threshold of three or more symptoms (derived from the childhood-onset category) to assign a diagnosis of ASAD.

The Depression Anxiety Stress Scale - DASS-21 [16] is a 21-item self-report measure that provides continuous scores on three subscales of depression, anxiety and stress, recorded for the past week. Items are scored from 0 (did not apply to me at all) to 3 (applied to me very much, or most of the time). High levels of severity on this measure are indicated by scores of 20, 14 and 26 or greater for depression, anxiety and stress, respectively. In the development of the measure, individual scales yielded Cronbach's alphas of 0.94 (depression), 0.87 (anxiety) and 0.91 (stress) [17].

The Work and Social Adjustment Scale - WSAS [18] is a self-report measure comprising subscales assessing functional impairment in the areas of work, home management, social leisure activities, private leisure activities (eg reading, gardening, etc) and close relationships. Items are rated on a Likert scale from 0 (affected "not at all") to 8 (affected "very severely, I never do these activities"). The measure has sound test-retest reliability and convergent validity [18]. A total score above 20 indicates high levels of functional impairment associated with a severe disorder; scores of 10 - 20 indicate significant impairment associated with mild to moderate level disorders; and scores below 10 are typical of a non-clinical population.

The Revised NEO Personality Inventory - NEO PI-R [19] is a self-completed scale measuring five personality traits: neuroticism, extraversion, openness, agreeableness and conscientiousness. Responses are coded on a five point scale ranging from "strongly disagree" to "strongly agree". Psychometric testing has supported the internal reliability of the scales. Normative data have been provided elsewhere [19]. In the present study, in order to facilitate statistical analysis, the personality dimensions were analysed as continuous indices.

The Separation Anxiety Symptom Inventory - SASI [20] is a 15-item self-report measure assessing separation anxiety symptoms retrospectively, based on experiences prior to 18 years of age. Items are scored from 0 to 3 on a frequency scale. The SASI has been shown to have sound internal (Cronbach's alpha = 0.88) and test-retest reliability over 24 months (intraclass correlation coefficient = 0.89). In the development of the measure, distributions were found to be skewed, a pattern adjusted for by applying a square root transformation. Hence, a raw score of 16 generates a transformed score of 4, whereas a score of 9 transforms into a score of 3. In past studies, mean transformed SASI scores of 4 or more have been associated with reports of past childhood separation anxiety disorder and/or school refusal, offering some evidence of the concurrent validity of the measure [21].

We also applied the DSM-IV-TR criteria for childhood separation anxiety disorder as reported retrospectively, in order to assess its occurrence prior to the age of 18 years.

### Statistical analyses

Three sets of analyses were undertaken for the whole sample and then by gender. Model 1 compared the conventional SCID-derived adult anxiety subcategories (ie PD, PD-AG, GAD and SP). In Model 2, those meeting criteria were assigned to the ASAD category, with all residual patients being grouped into a single category for comparison (ie ASADs and non-ASADs). Model 3 compared ASADs with all residual patients remaining in their initial diagnostic groups (ie PD, PD-AG, GAD, SP and ASAD).

Initial analyses indicated some variation in the number of comorbid anxiety and/or depressive disorders across primary anxiety categories (mean number of comorbid disorders associated with ASAD = 1.3, compared to 0.9 for PD, 1.0 for PD-AG, 0.9 for GAD and 0.9 for social phobia; *p *< .01 for all comparisons against ASAD). Since comorbidity generally is associated with severity of disorder [22], that factor could confound any comparisons we made, for example in contrasting ASADs with other anxiety categories in relation to indices of symptom severity and functional impairment. To address that issue, we entered the number of disorders (anxiety or depressive) per patient as a covariate in analyses involving continuous measures of the SASI, DASS, WSAS and NEO PI-R.

SPSS version 15 was used for all analyses [23]. Univariate analysis of variance was applied for continuous data with post hoc contrast testing. Categorical data were analysed using chi square tests. Significance levels were set at *p *< .01.

## Results

The results for the whole sample will be presented first, with gender-related differences reported thereafter.

### Model 1

The primary anxiety subcategories identified by the SCID were: PD (*n *= 121, 23% of total sample), PD-AG (*n *= 162, 31%), GAD (*n *= 135, 26%) and SP (*n *= 102, 20%). The mean age across all groups was 36 (*SD *= 12) years, with the SP group being younger (*p *< .01) compared to all other groups (see Additional file 2).

With the exception of those with SP, females predominated in all groups. Just under half the sample were married or in a cohabiting relationship (*n *= 244, 47%) except for the SP group, where only 22% (*n *= 22) were married, differing significantly from all other groups. Just over half the sample (*n *= 265, 51%) were employed, with PD-AGs being over-represented in the unemployed group (*n *= 72, 44%, *p *< .01) compared to those with PD and SP. Most were born in Australia and spoke English at home (all tests NS across groups for both indices). Additional file 3 shows that anxiety subcategories were similar in their reports of both indices of early separation anxiety.

Additional file 4 displays results for the DASS and WSAS, while Additional file 5 shows results for the NEO PI-R. Scores on these measures were not influenced by age. The anxiety subgroups returned similar scores on the DASS depression and stress scales. The PD and PD-AG groups scored higher on DASS anxiety compared to the GAD and SP categories. WSAS disability scores were higher for SPs and PD-AGs, primarily in the domains of work and social activities.

The SP group scored higher on the NEO PI-R subscale for neuroticism (see Additional file 5). SPs and PD-AGs scored lower than other groups on the extraversion and conscientiousness subscales.

### Model 2

The sample was then divided according to whether or not patients met criteria for ASAD. With the inclusion of that category, the total number of anxiety/depressive diagnoses assigned (primary and comorbid) was 921 or a mean of 1.8 per person. The numbers and percentages for each diagnosis were: ASAD = 207 (23%), PD = 108 (12%), PD-AG = 100 (11%), GAD = 195 (21%), SP = 132 (14%), and current depression = 133 (14%). Hence, the prevalence of ASAD assignments was roughly similar to that of GAD or the combined categories of PD/PD-AG. The proportion of primary diagnoses initially made on the SCID that were later assigned to the ASAD grouping, once formed, were: PD: *n *= 42 (35% of the initial PD group were re-assigned to the ASAD grouping); PD-AG: *n *= 80 (49%); GAD: *n *= 52 (39%); and SP: *n *= 33 (32%). A statistically greater number of those with an initial diagnosis of PD-AG was included in the ASAD grouping (*p *< .01). Females were overrepresented in the ASAD grouping.

The ASAD group had higher scores on all DASS subscales, on all disability scales of the WSAS (Additional file 4), and on NEO PI-R scores for neuroticism (Additional file 5). For depression analyses, we compared the ASAD group with a residual group where patients had 2 or more anxiety diagnoses, in order to broadly match the two groupings for levels of comorbidity in relation to other anxiety categories. No differences in rates of co-occurring current depression emerged from this comparison.

ASADs scored substantially higher on both indices of early separation anxiety (Additional file 3). We then divided the ASAD sample into those with probable childhood onset (SASI scores ≥ 4) and those with probable adult onset (<4). Three quarters of the ASAD sample (*n *= 151) had probable early onset and 52 were probable adult onset.

### Model 3

We then compared the ASAD grouping with specific subcategories of anxiety in the residual group. The ASAD and SP groups were younger, but statistically so only in relation to the GAD group. SP remained the only group with a minority of females (*n *= 31, 45% female), significantly so except in comparison with PD. As in Model 1, the SP group had more single and fewer married people compared to all other groups.

ASADs had higher DASS depression scores compared to all other groups, and higher stress scores than all groups but GADs. ASADs, PDs and PD-AGs reported higher levels of anxiety on the DASS than GADs and SPs. ASADs returned higher scores on both indices of early separation anxiety compared to all other groups (see Additional file 3). ASADs were more disabled on WSAS scales in relation to all other groups except on selective indices in relation to SPs.

ASADs and SPs scored higher on the NEO PI-R neuroticism scale than the other SCID anxiety categories. As in Model 1, the SPs scored lower on extraversion, while ASADs had the second lowest scores, although were significantly different only from the PD group.

### Analysis by gender

As indicated (Additional files 3, 4 and 5), the key analyses were also undertaken separately for males and females. Results largely replicated those for the total sample, although the differences between females with ASAD compared to their non-ASAD counterparts were more extensive than for the comparable analyses for males. We note however the smaller number of males (total sample: *n *= 359 females, *n *= 161 males), a factor that may have restricted statistical power for comparisons involving that gender.

One key finding that emerged from the gender-based analysis was that in both Model 1 and 3, SASI scores for the male-only social phobia group were significantly higher than the PD and PD-AG male groups (Additional file 3). In contrast, in the total sample and female-only analyses, the ASAD group alone scored significantly higher on the SASI. Minor differences also emerged on the NEO PI-R. Male ASADs returned statistically lower scores on extraversion and conscientiousness in Model 2 and were lower on agreeableness compared to the PD-AG group in Model 3. It should be noted, however, that all the relevant scores fell within the low to average range according to normative data [19].

## Discussion

The present data indicate that when ASAD was identified, that category comprised 23% of all diagnoses made in an adult anxiety clinic (taking into account that this figure includes both primary and comorbid disorders). The results are notable given that referral agencies and clinic staff did not explicitly identify ASAD as a distinct diagnostic category. Yet the severity of anxiety and depressive symptoms amongst ASADs was either as great or greater than other categories. Moreover, ASAD patients were more disabled in multiple domains of functioning, with the partial exception of those with SP. SPs in turn had a young age of onset, a high mean neuroticism score and low levels of extraversion, consistent with findings from epidemiological research [24,25].

In keeping with our previous studies [2,4], the data revealed an association between ASAD and early separation anxiety as measured by both indices. As indicated, a score of four (the square root transformation of the raw score) reported for the SASI has previously been associated with reports of clinically significant levels of separation anxiety in early life [21]. In addition, there is a high level of consistency in previous research showing a specific association between assignment to the ASAD category and elevated SASI scores [4,5,25].

Of interest, however, is the difference that emerged in the gender analysis: the relationship between high SASI scores and ASAD appeared to be specific for females, but amongst males those with both ASAD and social phobia returned elevated SASI scores. In addition, compared to the analyses for males, the differences on several indices were more extensive in women with ASAD compared to their female anxious counterparts. These data add to other evidence suggesting a gender difference in separation anxiety: separation anxiety is more common in females [3], familial and twin data support the possibility of a greater heritability factor amongst women [6], and the present data suggest that in females, separation anxiety is more likely to persist in an unaltered form over the course of development. In contrast, it may be that in males, early separation anxiety is a more general risk factor to the genesis of severely disabling anxiety in adulthood. Nevertheless, in drawing these inferences, it should be noted that the social phobia group may not have been representative of persons in the community with that disorder, amongst whom females outnumber males [26]. In general, females are more likely to seek treatment for social phobia [27], yet the clinic sample contained a small majority of males, in contrast to every other anxiety disorder. Hence, further research is needed to confirm the putative link between high SASI scores and social phobia in men, suggested tentatively by the present data.

Interpreting the distinction made between late and early onset cases based on retrospective SASI reports also requires caution. It is possible that patients with ASAD are prone to reporting analogous symptoms in early life. Only longitudinal studies commencing in childhood will be capable of addressing this issue critically. Hence the data can only offer tentative support for a developmental continuity theory which proposes that there may be a progression of separation anxiety symptoms from childhood into adulthood [4], a pattern that may be highly specific in females. If demonstrated to be correct, however, the continuity model will challenge the longstanding theory that early separation anxiety is specifically associated with risk to PD-AG in adulthood [28-30]. It is notable that previous studies testing the latter hypothesis did not include an adult form of separation anxiety disorder [31,32].

The pattern of comorbidity of ASAD with PD/PD-AG requires consideration. Definitional factors may account in part for the overlap, with several of the operational criteria of AG, as specified in DSM-IV-TR, being superficially similar to those of ASAD. For example, a reluctance to leave home is a characteristic of both disorders. Clinical experience suggests, however, that the underlying reasons for being housebound differ, with PD-AG patients seeking to avoid situations that trigger panic attacks, whereas the factors that motivate this behaviour in persons with ASAD relate to the need to maintain proximity to attachment figures.

The increased levels of neuroticism amongst ASADs and SPs suggest several possible interpretations. Early onset separation anxiety or social phobia may have a profound impact on character development, increasing the overall tendency towards lifelong worry and insecurity. Conversely, it is possible that anxiety-proneness in early life, a reflection of a possible heritable vulnerability, tends to express itself in symptom patterns that typically emerge in childhood and adolescence, namely SP and separation anxiety. The cross-sectional nature of the study does not allow us to reach a conclusion about the direction of causality in relation to these issues. The gender analyses suggested some personality differences in relation to males with ASAD. As indicated, however, scores for all the relevant indices fell within the low to average range for normative data, suggesting that the findings may not be of substantial clinical importance.

A greater recognition of the category of ASAD has important nosological implications. Debate continues as to whether the anxiety disorders should be conceptualised as categorical or dimensional [33]. Taxometric analyses have tended to support a dimensional pattern for most forms of anxiety, including adult separation anxiety [15,34]. From a dimensional perspective, it could be argued that symptoms of adult separation anxiety are an index of the overall level of severity of the disturbance suffered by anxious patients in general. It is plausible that as the severity of anxiety increases, persons with disorders such as PD-AG or GAD become more insecure, thereby generating a need to maintain proximity to attachment figures. That model might explain the pattern of comorbidity, symptom severity and disability displayed by those meeting criteria for ASAD in the present study. Nevertheless, epidemiological data [3] suggest that ASAD can occur on its own, at least in a minority of those with the diagnosis. Additionally, clinical data [1] indicate that where comorbidity exists, a historical review tends to suggest that separation anxiety symptoms preceded other symptoms of anxiety. That inference is supported by the endorsement of high levels of separation anxiety in childhood by patients with ASAD. As such, available evidence offers some support for the relative independence of ASAD as a form of adult anxiety.

Limitations of the study need to be considered. The methodology precluded our making judgments as to whether the diagnosis of ASAD was the primary condition requiring treatment. Future studies should apply a module for ASAD in the initial assessment, allowing clinicians to make decisions that include that category in assigning a primary diagnosis. Another limitation was that the diagnosis of ASAD was generated by self-report questionnaire [14], a different approach from that used for assigning other anxiety categories. Nevertheless, the measure of ASAD used has demonstrated a close concordance with a structured clinical interview based on the SCID format [14]. It seems likely that general practitioners screened out patients with serious medical conditions and comorbid anxiety, referring them to medical specialists including psychiatrists. Additionally, the study would have benefitted from the inclusion of information on participants' education levels, their use of psychotropic medications and any prior treatments. A previous report has indicated, however, the long and complex histories of treatment undergone by a substantial number of patients attending the clinic [35]. Controlling for the complex sequencing of past treatments for each patient was beyond the scope of the present study. Lastly, we note that patients with OCD and PTSD were excluded because of low numbers, a limitation of the study. Further research should examine for possible associations of ASAD with these two categories in a clinic setting.

## Conclusions

The present study suggests that the diagnosis of ASAD can be made in a substantial minority of patients attending an adult anxiety clinic. Those with ASAD had high levels of anxiety and depressive symptoms and were more disabled compared to those with other anxiety subcategories, with the partial exception of patients with social phobia. The findings suggest that future revisions of the classification system may need to acknowledge more explicitly that separation anxiety disorder can manifest throughout the life cycle. Clinicians should be better trained to identify ASAD both in primary and specialist care settings. In addition, there appears to be a pressing need to develop effective treatments that focus specifically on this disabling form of adult anxiety.

## Competing interests

The authors declare that they have no competing interests.

## Authors' contributions

DS played a major role in designing the study from its inception, directing the analyses and made a key contribution to writing and refining the article. CM, VM and RW contributed to the design and revision of the study. SR assisted in writing and revising the manuscript. All authors read and approved the final manuscript.

## Pre-publication history

The pre-publication history for this paper can be accessed here:

http://www.biomedcentral.com/1471-244X/10/21/prepub

## Supplementary Material

Additional file 1 **Appendix 1**. Algorithm of DSM-IV criteria applied to ASA-27 items. Click here for file

Additional file 2 **Table S1**. Demographic characteristics of patients grouped by their primary SCID diagnosis and after assignment to ASAD diagnosis. Click here for file

Additional file 3 **Table S2**. Mean scores on measures of developmental risk factors for adult separation anxiety, grouped by primary SCID diagnosis or ASAD diagnosis. Click here for file

Additional file 4 **Table S3**. Mean symptom severity and disability scores by primary SCID diagnosis and by ASAD grouping. Click here for file

Additional file 5 **Table S4**. Mean NEO PI-R personality scores, grouped by primary SCID diagnosis or ASAD diagnosis. Click here for file
