# Supplementary material for: The prevalence and correlates of adult separation anxiety disorder in an anxiety clinic
Source: BMC Psychiatry. 2010 Mar 10;10:21. doi: 10.1186/1471-244X-10-21 (PMC2846894; doi:10.1186/1471-244X-10-21)
Supplement: Additional file 1 — Appendix 1. Algorithm of DSM-IV criteria applied to ASA-27 items. [file 1471-244X-10-21-S1.DOC]

**Appendix 1**

Algorithm of DSM-IV criteria applied to ASA-27 items.

| DSM-IV Criteria for Separation Anxiety Disorder | ASA-27 questionnaire items |
| --- | --- |
| 1. Recurrent excessive distress when separation from home or major attachment figures occurs or is anticipated | Q11. Have you been especially concerned about where people close to you are going when you are separated from them, eg when you leave them to go to work or go out of the house?  Q14. Have you become very distressed when thinking about being away from people that are close to you?  Q18. Have you been worrying a lot about people you care about leaving you?  Q23. Have you been afraid that you would not be able to cope or could not go on if someone you cared about left you? |
| 1. Persistent and excessive worry about losing, or about possible harm befalling, major attachment figures | Q16. Have you been worrying a lot about people close to you coming to serious harm, eg meeting with a car accident, or suffering from a fatal illness? |
| 1. Persistent and excessive worry that an untoward event will lead to separation from a major attachment figure (eg getting lost or being kidnapped) | Q25. Have you been worrying a lot about possible events that may separate you from those close to you eg because of work requirements? |
| 1. Persistent reluctance or refusal to   go to school or elsewhere because of fear of separation | Q2. Have you experienced difficulty in staying away from home for several hours at a time?  Q6. Have you experienced extreme stress before leaving someone close to you when going away on a trip? |
| 1. Persistently and excessively fearful or reluctant to be alone or without major attachment figures at home or without significant adults in other settings | Q1. Have you felt more secure at home when you are with people that are close to you?  Q20. Have you tried to avoid being at home alone especially when people close to you are out? |
| 1. Persistent reluctance or refusal to go to sleep without being near a major attachment figure or to sleep away from home | Q12. Have you experienced difficulty in sleeping alone at night, eg is your sleep better if someone close to you is in the house?  Q13. Have you noticed that you are better able to go off to sleep if you can hear the voices of people you are close to or the sound of the TV or radio? |
| 1. Repeated nightmares involving the theme of separation | Q5. Have you suffered from nightmares or dreams about being separated from someone close to you?  Q15. Have you suffered from nightmares or dreams about being away from home? |
| 1. Repeated complaints of physical symptoms (such as headaches, stomach-aches, nausea, or vomiting) when separation from major attachment figures occurs or is anticipated | Q9. Have you experienced symptoms such as headaches, stomach-aches or nausea (or other) before leaving for work or other regular activity outside the home?  Q22. Have you found that you get anxious if you do not speak to people that are close to you on the telephone regularly, eg daily? |
