# Supplementary material for: The prevalence and correlates of adult separation anxiety disorder in an anxiety clinic
Source: BMC Psychiatry. 2010 Mar 10;10:21. doi: 10.1186/1471-244X-10-21 (PMC2846894; doi:10.1186/1471-244X-10-21)
Supplement: Supplementary file 2 [file 1471-244X-10-21-S2.DOC]

Table 1. Demographic characteristics of patients grouped by their primary SCID diagnosis and after assignment to ASAD diagnosis.

	Model 1				Model 2		Model 3				
	PD *N*=121	PD-AG *N*=162	GAD *N*=135	SP *N*=102	Non-ASAD *N*=313	ASAD *N*=207	PD *N*=79	PD-AG *N*=82	GAD *N*=83	SP *N*=69	ASAD *N*=207
Age *(SD)*	36 (13) $_{d}$	36 (12) $_{d}$	39 (13) $_{d}$	32 (10)	36 (12)	36 (13)	36 (14)	36 (11)	40 (13) $_{d, e}$	32 (10)	36 (13)
Duration of longest disorder (*SD*)	8 (12)	9 (10)	9 (11)	14 (11) $_{a, b, c}$	9 (11)	11 (11)	7 (11)	7 (8)	9 (11)	14 (12) $_{a, b, c}$	11 (11)
Females	89 (74%)$_{d}$	121 (75%)$_{d}$	103 (76%)$_{d}$	46 (45%)	199 (64%)	160 (77%) $_{f}$	52 (66%)	57 (70%)$_{d}$	59 (71%)$_{d}$	31 (45%)	160 (77%)$_{d}$
Marital status											
Single	43 (36%)	61 (38%)	34 (25%)	65 (64%)$_{a, b, c}$	125 (40%)	78 (38%)	30 (38%)	33 (40%)	18 (22%)	44 (64%)$_{a, b, c, e}$	78 (38%)
Married/ defacto	61 (50%)$_{d}$	77 (48%)$_{d}$	84 (62%)$_{d}$	22 (22%)	146 (47%)	98 (47%)	41 (52%)$_{d}$	36 (44%)$_{d}$	55 (66%)$_{b, d, e}$	14 (20%)	98 (47%)$_{d}$
Divorced/ widowed	17 (14%)	24 (15%)	17 (13%)	15 (15%)	42 (13%)	31 (15%)	8 (10%)	13 (16%)	10 (12%)	11 (16%)	31 (15%)
Employment status											
Employed	69 (58%)	74 (46%)	70 (52%)	52 (51%)	177 (57%)	88 (43%)	47 (60%)	43 (52%)	52 (63%) $_{e}$	35 (51%)	88 (43%)
Unemployed	33 (28%)	72 (44%) $_{a, d}$	45 (34%)	28 (28%)	95 (30%)	83 (40%)	21 (27%)	34 (42%)	21 (25%)	19 (28%)	83 (40%)
Age/ disability pension	6 (5%)	10 (6%)	13 (10%)	5 (5%)	16 (5%)	18 (9%)	4 (5%)	2 (2%)	7 (8%)	3 (4%)	18 (9%)
Student	12 (10%)	6 (4%)	6 (5%)	17 (17%) $_{b, c}$	24 (8%)	17 (8%)	6 (8%)	3 (4%)	3 (4%)	12 (17%)	17 (8%)
English spoken at home	92 (76%)	112 (69%)	96 (71%)	73 (72%)	226 (72%)	147 (71%)	57 (72%)	58 (71%)	62 (75%)	49 (71%)	147 (71%)
Born in Australia	82 (68%)	121 (75%)	93 (69%)	70 (69%)	218 (70%)	148 (72%)	50 (64%)	69 (84%)	53 (64%)	46 (67%)	148 (72%)

$_{a}$ = significantly greater than PD
$_{b}$ = significantly greater than PD-AG
$_{c}$ = significantly greater than GAD
$_{d}$ = significantly greater than SP
$_{e}$ = significantly greater than ASAD
$_{f}$ = significantly greater than non-ASAD
