# Supplementary material for: The prevalence and correlates of adult separation anxiety disorder in an anxiety clinic
Source: BMC Psychiatry. 2010 Mar 10;10:21. doi: 10.1186/1471-244X-10-21 (PMC2846894; doi:10.1186/1471-244X-10-21)
Supplement: Supplementary file 3 [file 1471-244X-10-21-S3.DOCX]

Table 2. Mean scores on measures of developmental risk factors for adult separation anxiety, grouped by primary SCID diagnosis or ASAD diagnosis.

	Model 1				Model 2		Model 3				
	PD mean *(SD)*	PD-AG mean *(SD)*	GAD mean *(SD)*	SP mean *(SD)*	Non-ASAD mean *(SD)*	ASAD mean *(SD)*	PD mean *(SD)*	PD-AG mean *(SD)*	GAD mean *(SD)*	SP mean *(SD)*	ASAD mean *(SD)*
SASI square root conversion* (total sample)	3 (1)	3 (1)	3 (2)	4 (1)	3 (1)	4 (1)$_{f}$	3 (1)	3 (1)	3 (2)	3 (1)	4 (1)$_{a, b, c, d}$
Females	3 (1)	4 (1)	3 (2)	4 (1)	3 (1)	4 (1)$_{f}$	3 (1)	3 (1)	3 (2)	3 (1)	4 (1)$_{a, b, c, d}$
Males	3 (1)	3 (2)	3 (2)	4 (1)$_{a, b}$	3 (1)	4 (2)$_{f}$	3 (1)	3 (1)	3 (1)	3 (1)$_{a, b}$	4 (2)$_{a, b, c}$
JSAD (total)	1 (2)	2 (2)	1 (2)	2 (2)	1 (1)	2 (2)$_{f}$	1 (2)	1 (1)	1 (1)	1 (1)	2 (2)$_{a, b, c, d}$
Females	1 (2)	2 (2)	2 (2)	2 (2)	1 (2)	2 (2)$_{f}$	1 (2)	1 (2)	1 (1)	0.5 (1)	2 (2)$_{a, b, c, d}$
Males	1 (1)	1 (1)	1 (2)	2 (2)	1 (2)	2 (2)$_{f}$	1 (1)	1 (1)	1 (2)	1 (2)	2 (2)$_{a, b}$

* square root of scores used as described in (15)

$_{a}$ = significantly greater than PD

$_{b}$ = significantly greater than PD-AG

$_{c}$ = significantly greater than GAD

$_{d}$ = significantly greater than SP

$_{e}$ = significantly greater than ASAD

$_{f}$ = significantly greater than non-ASAD
