# Supplementary material for: The prevalence and correlates of adult separation anxiety disorder in an anxiety clinic
Source: BMC Psychiatry. 2010 Mar 10;10:21. doi: 10.1186/1471-244X-10-21 (PMC2846894; doi:10.1186/1471-244X-10-21)
Supplement: Supplementary file 4 [file 1471-244X-10-21-S4.DOCX]

Table 3. Mean symptom severity and disability scores by primary SCID diagnosis and by ASAD grouping.

	Model 1				Model 2		Model 3				
	PD mean (*SD*)	PD-AG mean (*SD*)	GAD mean (*SD*)	SP mean (*SD*)	Non-ASAD mean (*SD*)	ASAD mean (*SD*)	PD mean (*SD*)	PD-AG mean (*SD*)	GAD mean (*SD*)	SP mean (*SD*)	ASAD mean (*SD*)
DASS Depression (total sample)	19 (12)	22 (12)	20 (11)	22 (11)	18 (11)	24 (11)$_{f}$	16 (10)	18 (12)	18 (10)	19 (12)	24 (11)$_{a, b, c, d}$
Females	19 (12)	22 (12)	19 (11)	20 (11)	16 (11)	24 (12)$_{f}$	15 (10)	19 (12)	17 (10)	18 (12)	24 (11)$_{a, b, c, d}$
Males	18 (12)	22 (12)	21 (10)	23 (11)	19 (11)	27 (11)$_{f}$	18 (11)	18 (11.0)	21 (8)	21 (12)	27 (11)$_{a, b}$
DASS Anxiety (total)	22 (11)$_{c, d}$	23 (11)$_{c, d}$	18 (10)	18 (10)	19 (11)	23 (10)$_{f}$	21 (11)$_{c, d}$	22 (12)$_{c, d}$	17 (11)	15 (10)	23 (10)$_{c, d}$
Females	22 (11)$_{c, d}$	23 (11)$_{c, d}$	18 (10)	17 (10)	17 (11)	24 (10)$_{f}$	21 (11)	21 (12)	16 (11)	16 (9)	23 (10)$_{c, d}$
Males	22 (11)	25 (10)$_{d}$	18 (11)	18 (10)	18 (11)	23 (9)$_{f}$	21 (11)	24 (11)$_{d}$	18 (11)	15 (10)	23 (10)$_{d}$
DASS Stress (total)	23 (9)	24 (10)	25 (10)	23 (10)	22 (10)	27 (10)$_{f}$	21 (8)	21 (10)	23 (10)	20 (10)	27 (10)$_{a, b, d}$
Females	24 (10)	24 (10)	25 (10)	23 (9)	21 (10)	28 (10)$_{f}$	21 (8)	21 (11)	23 (10)	21 (9)	27 (10)$_{a, b, c, d}$
Males	22 (8)	24 (10)	26 (11)	22 (10)	22 (10)	27 (9)$_{f}$	22 (8)	23 (10)	26 (10)	20 (11)	27 (10)$_{d}$
WSAS work (total)	4 (2)	4 (3)$_{c}$	3 (3)	5 (2)$_{a, c}$	4 (2)	5 (3)$_{f}$	3 (2)	4 (3)	3 (2)	5 (2)$_{a, b, c}$	5 (3)$_{a, b, c}$
Females	4 (3)	4 (3)$_{c}$	3 (3)	5 (2)$_{a, c}$	3 (3)	5 (3)$_{f}$	3 (3)	3 (3)	3 (3)	5 (2)$_{c}$	5 (3)$_{a, b, c}$
Males	4 (2)	5 (2)	4 (2)	5 (2)$_{a}$	4 (2)	6 (2)$_{f}$	4 (2)	4 (2)	4 (2)	5 (2)	6 (2)$_{a, c}$
WSAS home management (total)	3 (2)	3 (2)	3 (2)	3 (2)	3 (2)	4 (2)$_{f}$	3 (2)	3 (2)	3 (2)	3 (2)	4 (2)$_{a, b, c, d}$
Females	3 (3)	4 (2)	3 (2)	3 (2)	3 (2)	4 (2)$_{f}$	3 (2)	3 (2)	3 (2)	3 (2)	4 (2)$_{d}$
Males	3 (2)	3 (2)	3 (2)	3 (2)	3 (2)	4 (2)$_{f}$	3 (2)	3 (2)	3 (2)	3 (2)	4 (2)$_{a, b, d}$
WSAS social activities (total)	4 (2)	5 (2)$_{a, c}$	4 (2)	6 (2)$_{a, b, c}$	4 (2)	5 (2)$_{f}$	3 (2)	4 (2)	3 (2)	5 (2)$_{a, b, c}$	5 (2)$_{a, b, c}$
Females	4 (3)	5 (2)$_{c}$	4 (2)	5 (2)$_{a, c}$	4 (2)	5 (2)$_{f}$	3 (2)	4 (2)	3 (2)	5 (2)$_{a, c}$	5 (2)$_{a, c}$
Males	4 (3)	5 (3)	4 (2)	6 (2)$_{a, c}$	4 (2)	5 (2)$_{f}$	3 (2)	4 (2)	4 (2)	5 (2)$_{a, c}$	6 (2)$_{a, b, c}$
WSAS private leisure activities (total)	3 (2)	3 (2)	3 (2)	3 (2)	3 (2)	4 (3)$_{f}$	2 (2)	3 (2)	3 (2)$_{a, d}$	2 (2)	4 (2)$_{a, b, d}$
Females	3 (3)	3 (3)	3 (2)	3 (3)	3 (2)	4 (3)$_{f}$	3 (2)	3 (2)	3 (2)	2 (2)	4 (3)$_{a, b, d}$
Males	3 (2)	3 (2)	3 (2)	3 (2)	3 (2)	3 (2)	2 (2)	3 (2)	4 (2)$_{a}$	2 (2)	4 (2)$_{a}$
WSAS Total Score (total)	13 (8)	16 (8)$_{c}$	13 (7)	16 (7)$_{a, c}$	13 (7)	17 (8)$_{f}$	12 (8)	13 (7)	13 (7)	14 (6)	17 (8)$_{a, b, c}$
Females	14 (9)	16 (8)$_{c}$	13 (7)	16 (7)	12 (7)	17 (8)$_{f}$	12 (8)	12 (7)	12 (7)	14 (7)	17 (8)$_{a, b, c}$
Males	13 (8)	16 (8)	15 (7)	16 (6)	14 (7)	18 (7)$_{f}$	12 (6)	14 (7)	14 (6)	15 (6)	19 (7)$_{a, b, c}$

$_{a}$ = significantly greater than PD
$_{b}$ = significantly greater than PD-AG
$_{c}$ = significantly greater than GAD
$_{d}$ = significantly greater than SP
$_{e}$ = significantly greater than ASAD
$_{f}$ = significantly greater than non-ASAD
