# Supplementary material for: The prevalence and correlates of adult separation anxiety disorder in an anxiety clinic
Source: BMC Psychiatry. 2010 Mar 10;10:21. doi: 10.1186/1471-244X-10-21 (PMC2846894; doi:10.1186/1471-244X-10-21)
Supplement: Supplementary file 5 [file 1471-244X-10-21-S5.DOC]

Table 4. Mean NEO PI-R personality scores, grouped by primary SCID diagnosis or ASAD diagnosis.

	Model 1				Model 2		Model 3				
	PD mean *(SD)*	PD-AG mean *(SD)*	GAD mean *(SD)*	SP mean *(SD)*	Non-ASAD mean *(SD)*	ASAD mean *(SD)*	PD mean *(SD)*	PD-AG mean *(SD)*	GAD mean *(SD)*	SP mean *(SD)*	ASAD mean *(SD)*
NEO Neuroticism (total sample)	31 (8)	32 (8)	33 (7)	36 (7) $_{a, b, c}$	31 (8)	36 (7) $_{f}$	29 (8)	29 (8)	31 (7)$_{a}$	35 (7) $_{a, b, c}$	36 (7) $_{a, b, c}$
Females	31 (9)	32 (8)	33 (7)	36 (6)$_{a, b}$	30 (8)	36 (7)$_{f}$	27 (8)	29 (8)	31 (7)$_{a}$	35 (6)$_{a, b}$	36 (7)$_{a, b, c}$
Males	32 (8)	31 (8)	34 (7)	35 (7)$_{b}$	32 (8)	36 (7)$_{f}$	32 (8)	30 (8)	32 (6)	35 (7)$_{b}$	36 (7)$_{b}$
NEO Extraversion (total)	25 (7)$_{b, d}$	22 (7) $_{d}$	23 (7) $_{d}$	19 (7)	23 (7)	21 (8)	26 (6)$_{d, e}$	23 (7) $_{d}$	24 (6) $_{d}$	19 (7)	21 (8)$_{d}$
Females	25 (7)$_{b, d}$	22 (7)	24 (7)$_{d}$	19 (7)	23 (7)	22 (8)	26 (6)$_{d, e}$	23 (7)	24 (6)	21 (7)	22 (7)
Males	25 (7)$_{d}$	22 (6)$_{d}$	22 (7)	18 (7)	22 (7)$_{e}$	18 (8)	26 (6)$_{d, e}$	23 (5)$_{d, e}$	23 (7)$_{d, e}$	18 (7)	18 (7)
NEO Openness (total)	25 (6)	24 (6)	25 (7)	26 (6)	25 (6)	25 (7)	25 (6)	24 (6)	25 (6)	26 (6)	25 (7)
Females	26 (6)	24 (6)	25 (7)	26 (6)	25 (6)	25 (7)	26 (6)	24 (7)	25 (6)	26 (6)	25 (7)
Males	23 (6)	24 (6)	26 (5)	26 (6)	25 (6)	24 (7)	24 (6)	24 (5)	26 (5)	26 (7)	24 (7)
NEO Agreeableness (total)	31 (6)$_{d}$	30 (6)	30 (6)	29 (6)	31 (6)$_{e}$	30 (7)	31 (6)	32 (5)	31 (6)	29 (6)	30 (7)
Females	32 (6)	31 (6)	31 (6)	31 (6)	32 (6)$_{e}$	30 (6)	32 (6)	32 (5)	32 (6)	31 (6)	31 (7)
Males	31 (6)	29 (7)	29 (7)	27 (6)	29 (6)$_{e}$	27 (7)	31 (6)	31 (5)$_{e}$	29 (7)	28 (6)	27 (7)
NEO Conscientiousness (total)	30 (8) $_{d}$	28 (8)	31 (7)$_{b, d}$	27 (7)	30 (7)	28 (9)	31 (7)	28 (7)	31 (7)$_{e}$	29 (7)	28 (9)
Females	31 (9)	29 (8)	32 (7)$_{b, d}$	28 (7)	30 (8)	29 (9)	32 (8)	29 (8)	32 (7)	29 (7)	29 (9)
Males	29 (6)	27 (6)	30 (8)	27 (8)	29 (6)$_{e}$	25 (8)	29 (5)$_{e}$	28 (5)	30 (7)$_{e}$	28 (7)	25 (8)

$_{a}$ = significantly greater than PD
$_{b}$ = significantly greater than PD-AG
$_{c}$ = significantly greater than GAD
$_{d}$ = significantly greater than SP
$_{e}$ = significantly greater than ASAD
$_{f}$ = significantly greater than non-ASAD
